# 24h Urinary Sodium Excretion and Subsequent Change in Weight, Waist Circumference and Body Composition

**DOI:** 10.1371/journal.pone.0069689

**Published:** 2013-07-25

**Authors:** Sofus C. Larsen, Lars Ängquist, Thorkild I. A. Sørensen, Berit L. Heitmann

**Affiliations:** 1 Institute of Preventive Medicine; Bispebjerg and Frederiksberg Hospitals – a part of Copenhagen University Hospital, The Capital Region, Copenhagen, Denmark; 2 The Novo Nordisk Foundation Center for Basic Metabolic Research, Section on Metabolic Genetics, Faculty of Health and Medical Sciences, University of Copenhagen, Copenhagen, Denmark; 3 Research Centre for Prevention and Health, Glostrup University Hospital, Glostrup, Denmark; 4 The National Institute of Public Health, University of Southern Denmark, Copenhagen, Denmark; Paris Institute of Technology for Life, Food and Environmental Sciences, France

## Abstract

**Background:**

In the same period as the increasing obesity epidemic, there has been an increased consumption of highly processed foods with a high salt content, and a few studies have suggested that a diet with a high salt content may be associated with obesity.

**Objective:**

To investigate the association between 24 h urinary sodium excretion and subsequent change in body weight (BW), waist circumference (WC), body fat (BF) and fat free mass (FFM) among adults.

**Design:**

A longitudinal population study based on the Danish part of the MONICA project, with examinations in 1987–1988 and 1993–1994. Complete information on 24 h urinary sodium excretion along with repeated measures of obesity, as well as on potential confounders, was obtained from 215 subjects. Linear regression was used to examine the association between sodium excretion, as a measure of salt consumption, and subsequent changes in BW, WC, BF and FFM, and further evaluated by restricted cubic splines. Stepwise adjustments were made for selected covariates.

**Results:**

Neither the crude nor the adjusted models showed any statistically significant associations between sodium excretion and change in BW or WC. Likewise, we found no significant association between sodium excretion and change in BF and FFM in the unadjusted models. However, after adjusting for potential baseline confounders and the concurrent BW change, we found a significant increase in BF of 0.24 kg (P = 0.015, CI: 0.05 to 0.43) per 100 mmol increase in 24 h urinary sodium excretion (equivalent to 6 g of salt), during the 6-year study period. Moreover, during the same period, we found a significant association with FFM of −0.21 kg (P = 0.041, CI: −0.40 to −0.01).

**Conclusions:**

These results suggest that a diet with a high salt content may have a negative influence on development in body composition by expanding BF and reducing FFM.

## Introduction

For most of human existence we only consumed salt naturally contained in the food we ate, which was equivalent to a daily intake of less than 0.25 g salt pr. day. This changed when it was discovered that salt could be used to preserve food, making it an important product to ensure access to food through seasons with limited food availability [1]. In recent times, salt consumption has increased even further as we consume a wide variety of highly processed foods with a high salt content [2]. As the result, the average salt intake in most countries is nowadays approximately 9 to 12 g/d [1]. This increase in salt consumption has been shown to have a wide range of adverse effects on human health, primarily in relation to increase in blood pressure [3] and risk of cardiovascular disease [4].

Although an area with limited evidence, two cross-sectional studies also found that a diet with a high salt content was associated with body weight status [5], [6]. In addition, a recently published analysis from the DONALD (Dortmund Nutrition and Anthropometric Longitudinally Designed) study found a positive trend between urinary sodium excretion and increase in body weight and body fat percentage, among 364 children and adolescents [2]. An explanation for this could be that a high intake of salt stimulates appetite and thirst consequently increasing the total energy intake [3]. This explanation was supported by He et al. who found that a difference of 1 g/d in salt intake was associated with a difference of 27 g/d in sugar-sweetened soft drink consumption among children and adolescents [7]. Hence, it seems reasonable to assume that a potential effect of dietary salt on development of obesity may be the consequence of a poor or a high energy diet. However, neither inclusion of sugar-sweetened soft drinks nor total energy intake in the model changed the association found in the DONALD study [2], suggesting that other mechanisms may be operating. In favor of this, a cause not triggered by increased energy consumption has been proposed by Fonseca-Alaniz et al. who found that a high consumption of salt contributed to development of obesity among rats. This increase in adipose tissue was suggested to be caused by an increased capacity to incorporate glucose into lipids and that a higher lipogenic enzymatic activity may have promoted adipocyte hypertrophy and then excessive fat accumulation [8].

In the Danish MONICA (Monitoring Trends in Cardiovascular Disease) study, 24-hour urinary sodium was collected from a random subsample of 465 participants. The participants were subsequently followed for 6 years for development in obesity. Our aim was to investigate the association between 24-hour urinary sodium excretion and subsequent change in body weight (BW), waist circumference (WC), body fat (BF) and fat free mass (FFM).

## Materials and Methods

### Study population

The MONICA study was approved by the ethics committee for the Copenhagen County and is in accordance with the Helsinki II declaration on human rights. All participants in the study signed a letter of informed consent.

A random subset of 4,807 men and women born in 1922, 1932, 1942 and 1952 was selected among residents of 11 surrounding municipalities in the Copenhagen County. Of these, 3,608 participated in a health examination in 1982–83, which included measurements of BW, height and dietary intake, as well as blood sampling [9]. In 1987–88 and again in 1993–94 all surviving participants were invited to a second and a third health examination which included measurements of BW, height, WC and body composition measured by bioelectric impedance [10]. In 1987–88, a subset of 552 subjects was invited to a diet history interview and 435 attended both health examination and gave dietary information. Of these, 323 subjects returned complete 24 h urine samples (completeness measured with para-amino-benzoic-acid). Six years later, follow-up data on anthropometric measures were available for 262 of these subjects. For the present study, we further excluded participants with prevalent: Cancer (ICD-7 codes 140–148, 150–165, 170–181 and 192–207; n = 7), cardiovascular disease (ICD-8 codes 390–458 and ICD-10 codes I00-I99; n = 21) or self-reported diabetes (n = 2) and participants treated with diuretics (n = 17). A total of 215 subjects with complete data on 24 h urinary sodium and selected covariates measured in 1987–88 as well as anthropometric measures from the examinations in 1987–88 and 1993–94 were included in the current study.

### Collection and analysis of urine

24-hour urinary sodium samples were collected from participants examined in 1987–88 [11]. All subjects were instructed both orally and in writing on how to collect the urine samples. To monitor the completeness of the samples, each participant was given three tablets containing 80 mg para-amino-benzoic-acid, to be taken during the day of urine collection [12]. 24 h urinary sodium content was included in the analysis in units of mmol/24 h.

### Anthropometric measures

Height was measured to the nearest 0.5 cm, BW to the nearest 0.1 kg and WC horizontally midway between the lower rib margin and the iliac crest to the nearest 1 cm. All anthropometric measurements were taken in accordance with World Health Organization standards [13]. Body fat mass was measured by bioelectric impedance using a BIA-103 body-composition analyzer (RJL Systems, Detroit) following the manufacturer's instructions. As the precision of this method depends on how well the equation is adapted to the population, an equation was developed on a subset of 139 subjects, from the total population of 2,987 subjects examined in 1987–88, using a four compartment-model based on measurement of body water (dilutometry) and potassium counting (scintigraphy) as a reference [14]:
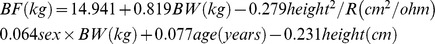
Here sex was coded as 1 for men and 0 for woman. Next, FFM was calculated by subtracting BF mass from BW and changes in BW, WC, BF and FFM were calculated as the difference between measures at health examination in 1987/88 and measures in 1993/94.

### Dietary information

The same trained dietician interviewed all the subjects using diet history interviews. Average daily intakes were calculated from responses describing the previous month. Data on meal patterns, dishes and food items were obtained with a pre-coded interview form. Quantities were assessed with food models, series of photographs, cups and measures. Finally, information was gathered about average weekly consumption of each type of alcohol (beer, wine and spirits). Calculations of nutrients were carried out with the DANKOST program, which is derived from the Danish food composition tables [12]. From this the daily intakes of total energy, dietary fat, protein and carbohydrate were calculated and included in the analysis as continuous variables (units: megajoule; MJ).

### Questionnaire data

Participants reported whether they had never smoked, were ex-smokers or current smokers. Smokers reported daily tobacco consumption, type of tobacco and how long they had smoked. This facilitated calculation of pack years (average number of packs smoked per day × years of smoking). Physical activity during leisure time was recorded in the following categories: 1) Almost completely inactive: sedentary activities such as reading, watching television and going to the movies. 2) Some physical activity: At least 4 hours weekly including for example walking, cycling, construction work, bowling and table tennis. 3) Regular hard activity at least 3 hours weekly, including for example swimming, tennis and badminton etc. or heavy gardening. 4) Hard activity: Elite sports such as swimming, soccer, badminton or long distance running several times a week. We merged group 3 and 4 because of insufficient numbers in group 4. Information on education level was classified with respect to having ≤7 years of education (yes/no). Finally we included information on the participants' age and gender, and the women reported whether they had entered menopause.

### Statistical analyses

All covariates were reported per tertile of baseline 24 h urinary sodium excretion with a p-value for trend across the tertiles based on a linear trend-parameter.

Linear regression was used to examine the association between 24 h urinary sodium excretion and changes in BW, WC, BF and FFM. All continuous variables were evaluated by model control (investigating linearity, consistency with a normal distribution and variance homogeneity). Initially the crude model was conducted, followed by a basic model which was adjustment for age, sex, educational level, smoking status, physical activity, alcohol consumption, height, baseline measure of outcome, and menopausal status for women. One at a time, we then added total energy intake, to assess whether there was an association independently of energy intake, and energy from macronutrients, to assess whether there was an association independently of the distribution of macronutrients. For the analysis with changes in WC, BF and FFM as outcome, we also added change in BW to assess whether these variables changed independently of BW. Finally, in the full model, we adjusted for all covariates – except macronutrients.

We further evaluated the associations using restricted cubic splines with 3 knots – positioned at the 10th, 50th and 90th percentiles of the present empirical distribution [15] – to allow for a non-linear association between 24 h urinary sodium and the outcome of interest. To examine whether or not any association seemed dependent on sex or age, we tested for interaction between sex and sodium, as well as age and sodium, by adding product terms to the model. Likewise, we tested if the association between 24 h urinary sodium excretion and change in WC, BF and FFM depended on the participants' weight change (weight gain/weight loss), by adding a product term to the model.

For statistical testing, p-values ≤0.05 were considered as statistically significant. The analysis was performed using the statistical software package Stata 9.2 (StataCorp LP, College Station, 2007).

## Results

Table 1 shows characteristics of anthropometric measures, dietary intake and other potential confounders by tertile of baseline 24 h urinary sodium excretion for the 215 included subjects. The table shows that male subjects generally consumed more salt than women. In addition to this, we found a significant trend towards increased height, weight- and lean mass status at baseline and follow-up with increased salt consumption and a significant trend between salt consumption and an increased intake of energy from all macronutrients.

**Table 1:** Baseline 24 h urinary sodium excretion, anthropometrics and covariates per tertile of baseline 24 h urinary sodium excretion.

	Tertiles of baseline 24h urinary sodium excretion			p trend^1^
	1^st^	2^nd^	3^rd^	
N	74	70	71	-
Sodium (mmol/day)	100 (60 to 130)	153 (132 to 180)	213 (183 to 335)	-
Baseline age, % ≤50 years	47.3	58.6	54.9	0.35
Sex (% women)	64.9	50.0	28.2	<0.001
**BW (kg)**				
Baseline	69.1 (52.0 to 90.0)	69.5 (50.4 to 94.0)	75.3 (54.0 to 94.8)	0.001
Follow-up	70.5 (54.3 to 91.4)	72 (50 to 96.8)	78.2 (56.1 to 106.7)	0.001
BW change	2.4 (−4.0 to 8.2)	1.9 (−4.6 to 11.3)	2.1 (−2.9 to 12.5)	0.78
**BF (kg)**				
Baseline	19.3 (9.7 to 32.8)	17.1 (8.7 to 31.2)	17.3 (7.3 to 33.2)	0.43
Follow-up	21.2 (12.6 to 36.8)	19.8 (10.7 to 33.6)	21.2 (9.0 to 38.9)	0.60
BF change	2.0 (−2.1 to 7.9)	2.3 to (−2.2 to 9.7)	2.3 (−1.3 to 9.6)	0.73
**FFM (kg)**				
Baseline	47.5 (37.2 to 60.9)	51.0 (37.8 to 66.6)	56.3 (40.2 to 74.0)	<0.001
Follow-up	47.0 (36.3 to 63.2)	48.3 (37.2 to 65.8)	57.6 (41.5 to 78.1)	<0.001
FFM change	0.001 (−2.57 to 2.14)	−0.24 (−2.10 to 3.68)	−0.16 (−2.58 to 2.40)	0.78
**WC (cm)**				
Baseline	79.5 (68 to 102)	83.2 (67.5 to 102.5)	88.0 (67.0 to 106.0	0.007
Follow-up	84.5 (71 to 102)	86.0 (67.0 to 102.0)	90.0 (72.0 to 110.0)	0.01
WC change	3.0 (−5.0 to 12.0)	2.5 (−6.5 to 10)	3.0 (−8.0 to 11.5)	0.66
Height (cm)	166 (156 to 181)	168 (155 to 183)	173 (161 to 193)	<0.001
Smoking (pack years)	1.2 (0 to 36.75)	9.5 (0 to 39)	8.0 (0 to 43.1)	0.19
Education, % ≤7 years	31.1	31.4	32.4	0.87
**Physical activity**				0.90
% Almost completely inactive	19.2	17.1	25.7	-
% Some physical activity	65.7	62.9	51.4	-
% Regular hard- & hard activity	15.1	20.0	22.9	-
Postmenopausal, % women	59.6	45.7	40.0	0.11
Total energy intake (MJ per day)^2^	7.3 (4.9 to 12.5)	8.4 (4.9 to 11.4)	8.2 (5.1 to 14.4)	0.002
Total dietary fat intake (MJ per day)	3.1 (1.8 to 5.8)	3.4 (1.5 to 5.1)	3.7 (2.1 to 6.1)	0.01
Total carbohydrate intake (MJ per day)	3.1 (1.7 to 5.2)	3.4 (1.8 to 5.2)	3.5 (2.0 to 7.5)	0.004
Total protein intake (MJ per day)	1.2 (0.8 to 1.7)	1.3 (0.8 to 2.0)	1.3 (0.9 to 2.1)	0.007
Alcohol (MJ per day)	0.2 (0 to 1.1)	0.2 (0 to 1.6)	0.2 (0 to 2.4)	0.03

Reported as median (5–95 percentiles) unless otherwise stated. Abbreviations: BW, body weight; BF, body fat; FFM, fat free mass; WC, waist circumference. ^1^ P-value for trend test based on linear trend-parameter. ^2^ Energy from alcohol is not included.

Changes in BW, WC, BF and FFM per 100 mmol increase in 24 h urinary sodium excretion (equivalent to 6 g of salt), during the six year period, between baseline and follow-up, are presented in Table 2. The crude model showed a change in BW of −0.02 kg (CI: −0.94 to 0.91) per 100 mmol increase in 24 h urinary sodium excretion. After including covariates in the basic model the association decreased to −0.18 kg (CI: −1.16 to 0.80). Neither total energy consumption nor energy consumption from macronutrients had any considerable influence on the size, direction or statistical significance of the association. Thus, in the energy-adjusted model, we found a change in BW of −0.15 kg (P = 0.77, CI: −1.12 to 0.83).

**Table 2:** Change in BW, WC, BF and FFM per 100 mmol increase in 24 h urinary sodium excretion with and without adjustment for covariates (estimate and 95% CI).

Included covariates	BW (kg)	WC (cm)	BF (kg)	FFM (kg)
Crude^1^	−0.02 (−0.94 to 0.91)	−0.23 (−1.22 to 0.76)	0.10 (−0.59 to 0.80)	−0.10 (−0.43 to 0.23)
Basic model^2^	−0.18 (−1.16 to 0.80)	0.18 (−0.81 to 1.18)	0.15 (−0.59 to 0.89)	−0.28 (−0.63 to 0.06)
Basic model + total energy intake	−0.15 (−1.12 to 0.83)	0.22 (−0.77 to 1.21)	0.17 (−0.57 to 0.91)	−0.27 (−0.62 to 0.07)
Basic model + macronutrients	−0.17 (−1.14 to 0.81)	0.20 (−0.79 to 1.19)	0.13 (−0.60 to 0.87)	−0.28 (−0.63 to 0.06)
Basic model + BW change	-	0.34 (−0.32 to 1.01)	0.24 (0.05 to 0.44)	−0.21 (−0.41 to −0.01)
Full model^3^	-	0.35 (−0.31 to 1.02)	0.24 (0.05 to 0.43)	−0.21 (−0.40 to −0.01)

Abbreviations: BW, body weight; BF, body fat; FFM, fat free mass; WC, waist circumference. ^1^ Crude: No adjustments. ^2^ Basic model: Adjusted for baseline age, sex, education level, smoking, physical activity, alcohol consumption, menopausal status for women, height and baseline measure of outcome. ^3^Adjusted for variables in the basic model + baseline total energy intake and change in BW between baseline and follow-up for the analysis of WC, BF and FFM.

In relation to change in WC, the crude model showed a change of −0.23 cm (CI: −1.22 to 0.76) per 100 mmol increase in 24 h urinary sodium excretion. After including covariates in the basic model the association increased to 0.18 cm (CI: −0.81 to 1.18). When we included BW change in the model, this association increased to 0.34 cm (CI: −0.32 to 1.01). Including total energy consumption or energy consumption from macronutrients in the model did not change the size of the association considerably and the association remained virtually the same after adjustment for all covariates in the full model, 0.35 cm (P = 0.30, CI: −0.31 to 1.02).

In relation to change in BF, we found change of 0.10 kg (CI: −0.59 to 0.80) in the crude model, while the basic model showed a change in BF of 0.15 kg (CI: −0.59 to 0.89). Including total energy consumption or energy from macronutrients in the model did not change the size of the association notably, whereas including BW change in the model resulted in a statistically significant increase in BF of 0.24 kg (CI: 0.05 to 0.44). This association persisted after adjustment for all variables in the full model, 0.24 kg (P = 0.015, CI: 0.05 to 0.43).

The crude analysis of change in FFM showed an association of −0.10 kg (CI: −0.43 to 0.23) per 100 mmol increase in 24 h urinary sodium. This association decreased to −0.28 kg (CI: −0.63 to 0.06) after inclusion of covariates in the basic model. Neither including total energy nor energy from macronutrients had any notable influence on the estimate. After including BW in the model the association decreased slightly but the confidence interval was also reduced causing a significant result of −0.21 kg (CI: −0.41 to −0.01). After including adjustment covariates in the full model we found a significant association of −0.21 kg (P = 0.041, CI: −0.40 to −0.01).

**Figure 1:**
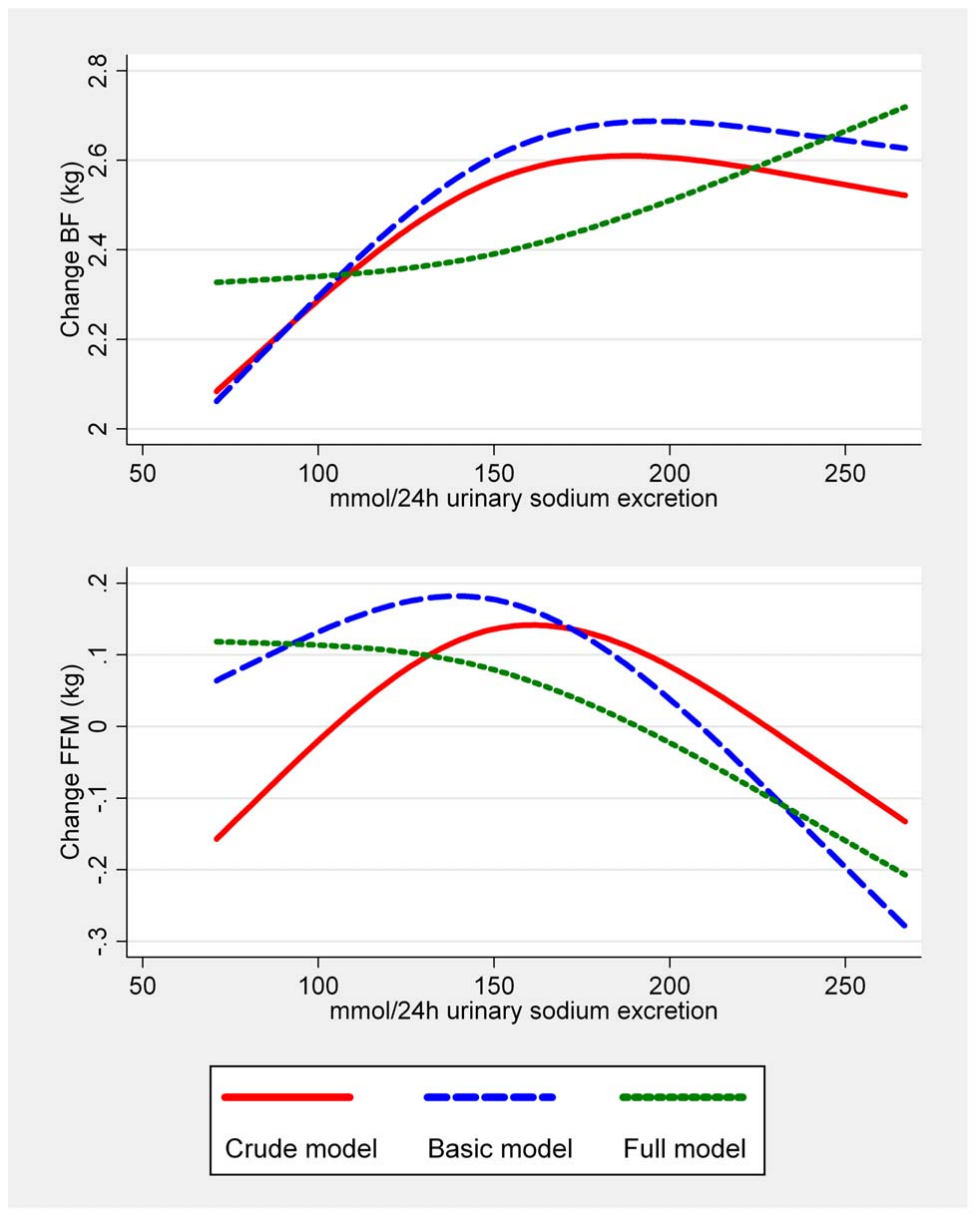
Restricted cubic splines of the association between 24 h urinary sodium excretion and change in BF and FFM. 3 knots positioned at the 10th, 50th and 90th empirical percentiles. Prediction lines presented over the empirical 5th-95th percentiles sodium excretion range. Abbreviations: BW, body weight; BF, body fat; FFM, fat free mass. The association between sodium excretion and change in BF and FFM are shown for a six-year period (1987–88 to 1993–94). Crude (Red line): No adjustments. Basic model (Blue line): Adjusted for baseline age, sex, education level, smoking, physical activity, alcohol consumption, menopausal status for women, height and baseline measure of outcome. Full model (Green line): Adjusted for variables in the basic model + baseline total energy intake and change in BW between baseline and follow-up.

The association between 24 h urinary sodium excretion and subsequent change in BW, WC, BF and FFM was further examined using restricted cubic splines. However, our tests for non-linearity were not significant and therefore we could not reject a linear relationship. Results are presented for analysis regarding changes in BF and FFM for all three models (Figure 1). In the crude model the data indicated a slightly bell shaped association with a maximum BF and FFM increase among those with a 24 h urinary sodium excretion around 140 and 175 mmol respectively. When all covariates were added in in the full model, we saw a transition towards a more linear relationship with the maximum increase in BF and decrease in FFM among those with the highest sodium excretion. This transformation towards a more linear relationship was caused by including the concurrent weight change in the model.

Finally, we tested for sex×sodium and age×sodium interactions (for all outcomes), as well as for BW change×sodium interaction (for analysis of WC, BF and FFM), but no significant results were observed.

## Discussion

In the present study, we found a positive association between 24 h urinary sodium and subsequent gain in BF and loss in FFM suggesting that a high salt consumption may play a role in development of obesity. Nevertheless, neither the crude nor the adjusted models showed any statistically significant associations between 24 h urinary sodium excretion and change in BW or WC. Likewise, we found no significant association between sodium excretion and change in BF and FFM in the unadjusted models. However, after adjusting for potential baseline confounders and the concurrent change in BW, we found a significant increase in BF of 0.24 kg (P = 0.015, CI: 0.05 to 0.43) per 100 mmol increase in urinary sodium, during the 6-year study period. Moreover, we found a significant association with FFM of −0.21 kg (P = 0.041, CI: −0.40 to −0.01), during the same period.

Our study has a number of strengths, including the longitudinal design with precise measures of exposure and outcomes, along with dietary history interviews conducted by the same trained dietician for all participants. Furthermore, we expect a good external validity as the data used in the present study is a random sample from the original MONICA cohort where there was a relatively high participation rate. Also, our study has a reasonably large collection of complete data on urine samples, anthropometry and body composition. Nevertheless, the study also has some limitations. In this regard we cannot reject the possibility of residual confounding from measures of dietary intake, as dietary information is always subject to some uncertainty. In studies of associations between dietary factors and changes in BW/composition the associations are often relatively weak. Consequently, it is possible that we have overlooked some associations due to lack of power. Moreover, urinary sodium is determined by recent intake, and therefore as variable as the actual salt consumption. The high variation was confirmed by Dyer et al. 1994 who reported an intraclass correlation of 0.44 for 24 h sodium excretions between two visits taken two weeks apart [16]. We could have improved the accuracy of our measure of exposure if we had performed repeated measurements of urinary sodium on the participants. However, inaccuracy in our exposure measurement cannot explain our findings, as the random error introduced by having an imprecise measure of exposure will lead to regression dilution. Consequently, the greater the variance in the sodium measurement, the closer the estimated slope of the observed associations should approach 0 instead of the true gradient. Hence, the observed associations were probably attenuated, rather than inflated.

Finally, sodium is essential in the body for controlling fluid balance and for maintaining blood volume and blood pressure within a normal range. Therefore a high salt consumption may induce body fluid retention and since bio impedance measurements predicts body composition partly from body water content this may have affected our results. Since salt retains water, one could argue that those with the highest consumption had the highest water retention. On the other hand, it is probably more likely to be changes in salt consumption that affects body fluid retention [1] and one study suggests that only an increase from a previous low salt consumption increases fluid retention [17]. However, it is difficult to say anything with certainty about the direction of this potential bias.

Several mechanisms could explain an association between sodium excretion and increase in BF. One explanation could be that a high intake of salt is the result of a high intake of high-energy foods with a high salt content, such as cheese and chips, consequently increasing the total energy intake. Likewise, studies have shown that a high intake of salt is associated with a high intake of soft drinks [7]. However, we included both total energy consumption and energy from macronutrients in our models, without altering the association, which could indicate that other mechanisms are operating. As a sensitivity analysis, we also performed the analysis with inclusion of soft drink consumption in the model. However, this did not change our findings. Similarly, the DONALD study found that increased urinary sodium was associated with increase in BF% among children and adolescents independent of their total energy intake and soft drink consumption [2]. Based on the study by Fonseca-Alaniz et al. 2007 this increase in adipose tissue could be caused by an increased capacity to incorporate glucose into lipids and by a higher lipogenic enzymatic activity hereby promoting adipocyte hypertrophy and then excessive fat accumulation [8]. The present study could not test these hypotheses but supports that the association does not seem to be mediated through energy intake.

The results of our study suggest that a diet with a high salt content may have a negative influence on development in body composition by expanding BF and reducing FFM. Salt consumption was not associated with change in BW or WC in this study, possibly because salty food affects BF and FFM in opposite directions. However, the absence of significant findings in this regard might also be due to lack of power. Hence, larger samples with measures of 24 h urinary sodium, along with repeated measures of anthropometry and body composition, are needed to clarify the association between salt consumption and development of obesity, along with further studies of the biological mechanisms underlying the possible relationship.
